# Million dollar personality: a systematic literature review on personality in crowdfunding

**DOI:** 10.1007/s11301-021-00242-9

**Published:** 2021-11-10

**Authors:** Julia Neuhaus, Andrew Isaak, Denefa Bostandzic

**Affiliations:** 1 Manchot Graduate School, Competitiveness of Young Enterprises (WEJU), Heinrich-Heine-University of Düsseldorf, Universitätsstrasse 1, 40225 Düsseldorf, Germany; 2 Corporate Finance, Witten/Herdecke University, Alfred-Herrhausen-Straße 50, 58448 Witten, Germany

**Keywords:** Literature review, Personality, Crowdfunding, Five-factor model, Dark triad, D80, D91, G30, G41, L14, L26, O30

## Abstract

Expressed personality traits can play a pivotal role in convincing investors in crowdfunding. Our study answers the research question: What is the current body of knowledge regarding the relationship between personality factors and crowdfunding success and where are knowledge gaps where the literature is silent? In our literature review, we therefore analyze and categorize (1) the results provided by quantitative studies on the relationship between the personality of entrepreneurs and crowdfunding success and (2) the research gaps identified by the authors investigating personality in crowdfunding. We find that studies investigating the entrepreneur's personality, i.e. the Big Five, other baseline personality traits (self-efficacy, innovativeness, locus of control, and need for achievement) and the Dark Triad, find positive relationships between openness and crowdfunding success, while narcissism shows an inverted u-shaped relationship with crowdfunding success across articles. However, the effects of other personality traits on crowdfunding success are largely inconclusive. Further, we identify four main gaps in the literature. First, future studies should examine non-linear relationships between expressed personality traits and crowdfunding success. Second, there is a need for more studies that employ different methods like qualitative or mixed-method approaches. Third, replication studies in similar and different contexts are urgently needed. Fourth, a plurality of personality perspectives would strengthen future research (e.g., investor perspective, third party perspective). To our knowledge this is the first literature review of personality traits in crowdfunding. Our work aims to enrich our understanding of individual-level components in the underexplored alternative finance market.

## Introduction

Young firms face the challenge of acquiring early stage venture capital (Drover et al. 2017) which more than doubles their chances of survival (Puri and Zarutskie 2012). To finance their venture, entrepreneurs increasingly face a number of options outside of traditional venture capital funding or business angel investments. One example of such alternative financing methods is crowdfunding, which opens new pathways for young firms to raise capital in a less regulated way than via classical funding instruments (Cumming et al. 2019b). Crowdfunding presents a financing method in which firms acquire capital from a crowd of individuals via an open call (Belleflamme et al. 2010). Entrepreneurs turn to crowdfunding when they need financial assistance to realize a project. Via crowdfunding, entrepreneurs can also acquire customers and validate their business models or ideas at an early stage while simultaneously retaining a high degree of independence from individual investors. Types of crowdfunding include borrowing money online for investments (lending-based), offering products or rewards for pre-sale (reward-based), collecting donations to realize charitable projects (donation-based), or selling equity shares of a company to a crowd of investors (equity-based). The types of crowdfunding significantly differ from each other. For example, equity crowdfunding gears towards long-term investments, whereas other types of crowdfunding typically involve pre-selling, short-term loans, or donations regarding future projects. Similarly, entrepreneurs seeking equity crowdfunding are in a somewhat similar stage to those that receive classical venture capital or angel financing, as these settings both involve a (long-term) stake in the venture. This similarity does not hold for most other crowdfunding forms.

A growing stream of literature investigates factors that lead to successful crowdfunding (Wiklund et al. 2011). Authors find that several “hard facts” such as the target investment amount, the number of investors/backers to date, provided roadmaps, Facebook shares, or the location of a company impact the outcome of a crowdfunding campaign (Ahlers et al. 2015; Bertrand and Schoar 2003; Bi et al. 2017; Block et al. 2018; Chan and Parhankangas 2017; Courtney et al. 2017; Davis et al. 2017; Janku and Kucerova 2018; Prodromos et al. 2014). “Softer factors” that include media richness (e.g., use of photos and videos), third-party endorsement, and campaign updates can also drive the funding process (Courtney et al. 2017; Wang et al. 2020). In addition, individual-level factors are critical for crowdfunding success. For example, entrepreneurs’ education and professional background, previous funding experience, and gender or ethnic background can influence the crowd’s contributions to a given campaign (Allison et al. 2017; Barbi and Mattioli 2019; Courtney et al. 2017; Moleskis et al. 2019; Younkin and Kuppuswamy 2018).

Within this stream, a unique discourse relates to the entrepreneur's personality. Personalities describe the unique combinations of traits that form people's individual character. In line with the entrepreneurship field in general, research in crowdfunding has also begun to study the impact of personality on funding success. Two studies examine the influence of the Big Five personality traits on reward-based crowdfunding success on the website Kickstarter (Gera and Kaur 2018; Thies et al. 2016). Further, Bollaert et al. (2019) research indicates a negative impact of narcissistic personality traits on funding success, while other authors find inconclusive relations of narcissistic rhetoric to crowdfunding success depending on the compliance with other characteristics of the entrepreneur (Anglin et al. 2018b). Regarding hubris and charisma, researchers have found that entrepreneurs perceived as scoring high on these traits are more successful in raising funds (Sundermeier and Kummer 2019). Moritz et al. (2015) argue that perceived sympathy, openness, and trustworthiness are essential in reducing information asymmetries (e.g., where one party knows more than the other and could exploit this information supremacy) between entrepreneurs and investors in the crowdfunding context.

As an alternative method of financial resource acquisition, crowdfunding is of special interest for entrepreneurship research (Landström and Harirchi 2019), especially when combined with the “most promising topical areas in entrepreneurship research” (Kuckertz and Prochotta 2018, p. 3), e.g., entrepreneurial behavior and psychology. Although promising, crowdfunding does not come without challenges for entrepreneurs seeking capital and particularly for investors when trying to discern entrepreneurs’ chances of success. On the one hand, investors face increased information asymmetries than they would in other funding types (Cumming et al. 2019a). These arise from reduced disclosure requirements for fund-seeking entrepreneurs (Cumming et al. 2019a), the use of new media tools, and the lack of opportunity to directly question campaign initiators. Such circumstances increase the need for cognitive shortcuts to make investment decisions. These are based (among others) on impressions of entrepreneurs’ personality and used, for example, to access the entrepreneur's capability to lead a successful venture. For entrepreneurs, on the other hand, funds are not acquired via direct interaction but through means of computer-mediated communication (Pollack et al. 2021). Investments are mediated by online fundraising platforms where personality is displayed and perceived in a very different way than in traditional and interpersonal settings (e.g., with an angel investor or loan agent). For entrepreneurs in the context of crowdfunding, knowing which personality displays convince the crowd to invest in their campaigns is of particular practical relevance, as it can shape investor perception and therefore campaign success. In crowdfunding, the personality impression perceived by investors is literally worth up to a million dollars (JOBS Act; (Ahlers et al. 2015)), inspiring the title of this paper.

Although a growing body of literature summarizes and evaluates crowdfunding success factors, personality plays no role in these reviews. To our knowledge, no previously published literature review focuses on personality factors in crowdfunding, although the implications both for practice (as explained above) and for the scientific community are essential. Combining the representative findings on crowdfunding and personality from disparate studies into one literature review would focus future research on relevant gaps and broaden the impact of this field. Additionally, identifying areas where the results from crowdfunding are generalizable to other forms of entrepreneurial financing would create the opportunity to transfer implications from crowdfunding, with its easy accessibility and high sample sizes, to other areas where research is scarce due to difficulties to access data (e.g., business angel financing) (Cumming et al. 2019a, b). We address this gap by examining the following research question: What is the current body of knowledge regarding the relationship between personality factors and crowdfunding success, and where are knowledge gaps where the literature is silent?

Our study finds a trend towards more research on entrepreneurial personality in crowdfunding and a tendency to employ software-based narrative methods and questionnaires. We identified four main gaps that should be addressed by future research studies. First, future quantitative studies should examine nonlinear (e.g., quadratic) relations between expressed personality traits and crowdfunding outcomes. Second, future studies should employ different methods e.g., mixed-methods approaches in order to validate existing narrative methods, such as by combining them with questionnaires. Third, authors should conduct replications in highly similar settings to strengthen results as well as in different contexts, e.g., crowdfunding types, to explore different effects of personality. Fourth, studies are required that investigate not only the personality of entrepreneurs but change/flip the perspective and also investigate the personality of investors and how they interact during the crowdfunding process.

In the following, we first describe the conceptual background of personality constructs and the chosen methodology, as well as our analysis of the selected literature. Finally, we highlight commonalities, differences and gaps, in addition to implications and suggestions for future research.

## Conceptual Background on Personality in the Entrepreneurial Context

The personality of an individual is the basis that effects a person’s decisions and behavior in everyday life situations as well as in the economic aspects of life (McAdams and Pals 2006; Rauch and Frese 2014). The broad concept of personality includes a range of aspects from abilities such as different forms of intelligence, motives, attitudes up to a person’s characteristics and temper (Brandstätter 2011). Taken together, personality can be seen as the foundation for individual differences between humans (Mairnesse et al. 2007). Studies suggest that personality is an underlying system that develops until the age of 30 and then stays stable over adolescent life (Costa and McCrae 1988). In the entrepreneurship literature, authors investigate a wide variety of personality aspects.

The personality theory most frequently investigated in entrepreneurship is the Big Five Personality Theory from psychology (e.g., Brandstätter 2011; Kerr et al. 2017; Mueller and Thomas 2001; Rauch and Frese 2014). Research in entrepreneurial finance finds effects of the Big Five on business angel syndication, investment management, and loss aversion in the financial domain (Block et al. 2019; Boyce et al. 2016; Mayfield et al. 2008). The concept focuses on five key traits: First, openness, when strongly expressed, is a driver for the need for variety and intellectual curiosity (Costa Jr and McCrae 1995). People that rate high on openness seek new experiences. In a business-related context, people with high openness ratings are socially skilled. Scientists suggest that they are good salespeople and have managerial skills (Almlund et al. 2011). People who rate low on openness are risk-averse (Almlund et al. 2011). Researchers associate openness with intelligence and creativity, but also with negative aspects such as sensation-seeking and a tendency to question authority (Costa Jr and McCrae 1995). Second, conscientiousness relates to striving for achievement, hard work, dutifulness, and self-discipline (Almlund et al. 2011; Bozionelos 2004). In the business context, conscientiousness is a predictor for career success, job performance, and wages (Almlund et al. 2011; Hogan and Ones 1997; Judge et al. 1999). Third, extraversion is associated with sociability, optimism, ambition, positive emotionality, cheerfulness, dominance, and excitement seeking (Barrick et al. 2001; Bozionelos 2004; Watson and Clark 1997). High scores in extraversion predict effective job performance, the likelihood to reach a leadership role, and wages (Almlund et al. 2011; Barrick and Mount 1991; Bozionelos 2004; Ciavarella et al. 2004; Judge et al. 1999). Fourth, agreeableness is a trait often summarized as warmness. People with high scores on agreeableness tend to be altruistic, friendly, flexible, courteous, forgiving, modest, and trustworthy (Almlund et al. 2011; Barrick et al. 2001; Bozionelos 2004). Studies demonstrate a negative relationship between agreeableness and career success or work involvement (Bozionelos 2004). Fifth, neuroticism (also referred to as emotional instability) is related to the experience of negative emotions, insecurity, low goal-orientation, and low self-esteem (Almlund et al. 2011; Bozionelos 2004). Research also finds negative associations between neuroticism and job search efforts, work performance, performance motivation, and extrinsic success (Almlund et al. 2011; Judge and Ilies 2002).

Other baseline key personality traits frequently studied in entrepreneurship (aside from the Big Five) are self-efficacy, innovativeness, locus of control, and need for achievement (Kerr et al. 2017; Rauch and Frese 2014), explained hereafter. First, self-efficacy as part of the personality is of particular interest regarding entrepreneurs as it describes a person's inclination to see themselves as capable of performing actions and aligning themselves with self-set goals (Chen et al. 1998; Rauch and Frese 2014). Overcoming failure can also be counted as self-efficacy (Harburg et al. 2015). Second, innovativeness is strongly linked to a person's ability to engage in new things. Innovative people are those in a society who adapt to change faster than the average (Manning et al. 1995; Rogers and Shoemaker 1971). Since innovativeness is a prerequisite for innovation, it is a crucial personality component in entrepreneurship. Third, locus of control is closely linked to a person's belief in their ability to determine their destiny (Hoffman et al. 2003). Researchers differentiate between external and internal locus of control. An external locus of control refers to when people perceive their future to be shaped by their environment and not by their own actions. In general, founders tend to have an internal locus of control, which refers to situations where people are convinced that they can shape their future by their actions and decisions (Rotter 1966). Fourth, the need for achievement is a personality factor that goes back to David McClelland's Motivation Theory (Johnson and McClelland 1984). A high need for achievement describes people who are not satisfied with routine tasks but strive for challenges and continuous improvement (Rauch and Freser 2014). They take responsibility for the results they achieve and demand feedback for their actions. Many studies highlight the relevance of this trait for founders (Rauch and Frese 2007), as it can influence venture size and growth (Lee and Tsang 2001).

A personality aspect of increasing interest to researchers is narcissism (Bollaert et al. 2019; Butticé and Rovelli 2020). Narcissistic individuals are generally perceived as arrogant and self-centered. They usually have an elevated image of their achievements and react with offense or even aggression when questioned (Miller et al. 2010). On the other hand, narcissism can also have positive effects, e.g., on self-confidence and self-respect, if not overly expressed (Paulhus and Williams 2002). Therefore, these characteristics are clearly relevant for entrepreneurs. Narcissism is one of three characteristics summarized as the “Dark Triad” (Paulhus and Williams 2002) which refers to the three socially aversive traits narcissism, Machiavellianism, and psychopathy. These traits reflect self-promotion, emotional coldness, and aggressive behavior in a person's character (Paulhus and Williams 2002). Focusing on manager characteristics, the dark triad and, in particular, narcissism diminish the positive effect of entrepreneurial orientation and thereby negatively influence firm performance (Bouncken et al. 2020; Engelen et al. 2016). Narcissism and psychopathy are officially classified as psychological disorders in the U.S. and Europe (e.g., in DSM 4 and 5) (Furnham et al. 2013). However, the entrepreneurial literature uses them to describe personality aspects that tend towards the clinical definition but do not necessarily fit this pathological description of narcissism.

In the following section, we focus on those traits most frequently addressed in entrepreneurship and introduced above (Kerr et al. 2017; Mueller and Thomas 2001; Rauch and Frese 2014). These are the Big Five personality model, the additional baseline traits innovativeness, self-efficacy, locus of control, need for achievement, and the Dark Triad.

## Methodology

### Data collection

To answer our research question, we followed the guidelines set forth by Fisch and Block (2018). Therefore, we began by screening the existing literature. We collected the articles for this review in May of 2021, allowing us to take a snapshot of the literature on personality in crowdfunding. To obtain a comprehensive overview of literature on the topic, we did not limit our search to specific journals (Webster and Watson 2002). Instead, we rely on the leading databases of the field, such as EBSCO Host, Scopus, and Web of Science. Our literature search involved four steps:

First, we searched the databases. For each of these we used the closest corresponding filter criteria available (abstract search in EBSCO Host, abstract and title search in Scopus, and topic search in Web of Science). For the search we combined the term “crowdfunding”, “P2P lending”, or “peer-to-peer lending” and one of the following terms on personality: “personality”, “big five”, “openness”, “conscientiousness”, “extraversion”, “agreeableness”, “neuroticism”, “dark triad”, “narcissism”, “self-efficacy”, “innovativeness”, “locus of control”, and “need for achievement”. Table 1 provides further information on the search strings employed and the respectively resulting number of articles. The initial search generated 20 unique EBSCO host articles, 65 unique Scopus articles, and 45 unique Web of Science articles resulting in 81 unique articles over all three platforms (removing duplicates).

**Table 1:** Initial Search

Search Term	EBSCO^1^	Scopus^1^	WoS^1^
crowdfunding AND personality	5	15	14
crowdfunding AND “big five”	1	4	2
crowdfunding AND openness	7	17	7
crowdfunding AND conscientiousness	0	4	1
crowdfunding AND extraversion	1	5	2
crowdfunding AND agreeableness	0	4	1
crowdfunding AND neuroticism	0	3	1
crowdfunding AND “dark triad”	0	0	0
crowdfunding AND narcissism	2	4	4
crowdfunding AND self-efficacy	2	8	7
crowdfunding AND innovativeness	7	25	14
crowdfunding AND “locus of control”	1	0	1
crowdfunding AND “need for achievement”	0	0	0
(“peer-to-peer lending” OR “P2P lending”) AND personality	0	1	3
(“peer-to-peer lending” OR “P2P lending”) AND “big five”	0	0	0
(“peer-to-peer lending” OR “P2P lending”) AND openness	1	2	1
(“peer-to-peer lending” OR “P2P lending”) AND conscientiousness	0	0	0
(“peer-to-peer lending” OR “P2P lending”) AND extraversion	0	0	0
(“peer-to-peer lending” OR “P2P lending”) AND agreeableness	0	0	0
(“peer-to-peer lending” OR “P2P lending”) AND neuroticism	0	0	0
(“peer-to-peer lending” OR “P2P lending”) AND “dark triad”	0	0	0
(“peer-to-peer lending” OR “P2P lending”) AND narcissism	0	0	0
(“peer-to-peer lending” OR “P2P lending”) AND self-efficacy	0	0	0
(“peer-to-peer lending” OR “P2P lending”) AND innovativeness	0	2	0
(“peer-to-peer lending” OR “P2P lending”) AND “locus of control”	0	0	0
(“peer-to-peer lending” OR “P2P lending”) AND “need for achievement”	0	0	0
Unique papers per Database	20	65	45
Unique papers across Databases		81	

^1^Search results from the 20.05.2021

In a second step, we screened all retrieved articles and included them in our review based on subject matter fit. We therefore excluded all articles with no clear focus on crowdfunding or on personality. We also exclude those studies that solely mention personality, but do not actually include one or more personality constructs or crowdfunding in their research. In case of personality this exclusion criterion is complicated to assess because researchers often use the term personality to describe personal characteristics (e.g., optimism) rather than concrete personality constructs (e.g., agreeableness). To differentiate the papers that actually explore personality constructs in the context of crowdfunding from those that do not, we asked ourselves the following three questions while examining the papers:

(1) Do the search terms appear within the title, abstract, or keywords of the paper, or is it a mismatched result (i.e., where the terms do not really appear as expected)? For example, we excluded Borst et al. (2018) as none of our personality-related terms were mentioned within the title, abstract, or keywords (“From friendfunding to crowdfunding: Relevance of relationships, social media, and platform activities to crowdfunding performance”).

(2) Is personality/crowdfunding a *core concept* of the paper or just used as an example to research a related topic? For example, we excluded Gruda et al. (2021) as crowdfunding is just a concept to which the paper's results are compared (i.e. “We discuss and compare our findings to previous work on narcissism and crowdfunding.” (Gruda et al. 2021, p. 1)); another example is the exclusion of Wang et al. (2017) who investigate sentiments rather than personality (“The study proves that positive sentiment in the blurb and detailed description promotes the successful campaigns” (Wang et al., 2017, p. 2)).

(3) Is the construct related to a person/group? For example, we excluded Ceballos et al. (2017) as product innovativeness is not a characteristic of the entrepreneur (“the innovativeness of a project, […] can positively affect crowdfunding achievement.” (Ceballos et al. 2017, p. 79)).

For the 81 articles, two researchers assessed the relevance of each article by screening the title, abstract, and keywords and by employing the three questions as additional fit criteria to decide on the relevance for the literature review. If the title, abstract, and keywords were insufficient to assess whether or not the article should be included in the review, the whole paper was read to reach a clear conclusion (8 articles, e.g., Shin and Lee 2020). This rating method was conducted by two authors independently. In cases of disagreement (12 articles, e.g., Tseng 2020), the articles were discussed until a consensus was reached. This procedure led to the inclusion of 25 (out of 81) articles.

In the third step, we performed subsequent forward and backward searches, using both the reference lists of the articles and Google Scholar. We used the aforementioned criteria to assess the relevance of the retrieved articles, yielding three additional articles for our data set, for a total of 28.

As the last step, we also examined other literature reviews on crowdfunding. In these, however, the focus was mostly on general success factors (Alegre and Moleskis 2019; Bouncken et al. 2015; Butticé et al. 2018; Cai et al. 2021; Dalla Chiesa and Handke 2020; Iurchenko 2019; Jovanović 2019; Kaartemo 2017; Mochkabadi and Volkmann 2020; Moleskis and Alegre 2018; Moritz and Block 2016; Salido-Andres et al. 2020; Shneor and Vik 2020; Zhao and Ryu 2020). Overall, personality was only mentioned as a success factor in one of the reviews (Butticé et al. 2018), which further illustrates the necessity of our work.

**Fig. 1:**
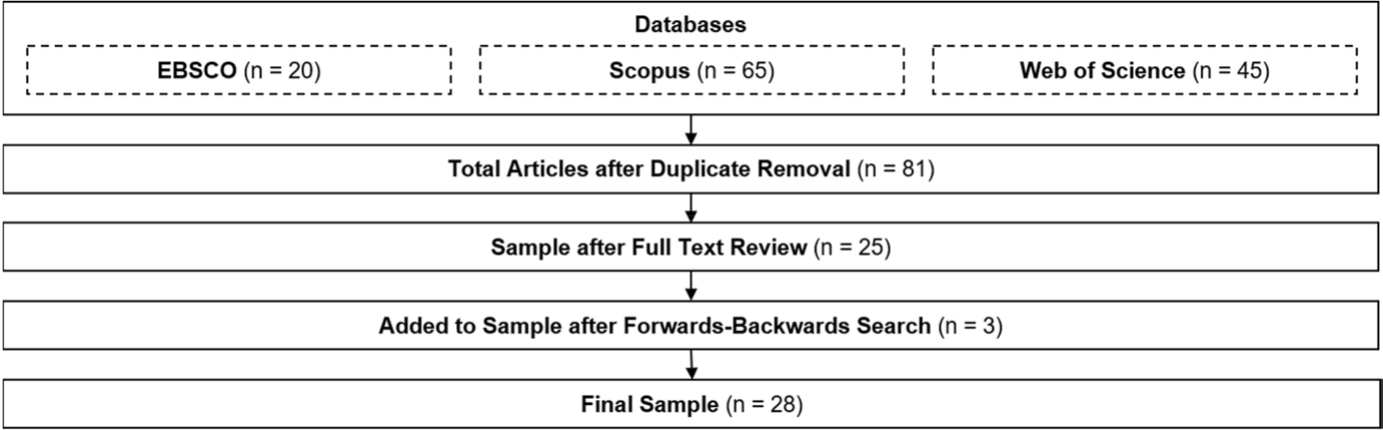
Systematic Data Collection Process

For our review, we only included articles written in English and published in peer-reviewed academic journals, research compilations or conference proceedings. The only exception to this was a dissertation on the Dark Triad by an expert in the field (Creek 2018). Overall, our literature screening resulted in a collection of articles that very clearly examine crowdfunding and personality with a particular emphasis on the personality aspects we included in our search terms. The steps of the literature search and selection are summarized in Fig. 1 below.

### Data analysis

After carefully screening the articles, we decided on a topic-centered analysis. Therefore, we first collected classical descriptive data on the articles in our dataset (e.g., publication date, outlet, research method). We also identified and recorded topic-specific descriptive data; for example, we determined the crowdfunding type described in the articles (reward-based, equity-based, lending-based, or donation-based), categorized the theoretical approach (e.g., signaling theory, social identity theory), the methodologies utilized (e.g., questionnaire, narrative analysis, etc.), and the variables employed (e.g., Big Five personality, innovativeness) in more detail. We also identified the authors' perspectives on their investigation and categorized these as campaign owner-centered, investor-centered, or as a hybrid approach (Table 2). After the articles were categorized by one author using the citation management software Citavi, they were reviewed by another researcher without significant discrepancies after discussion.

**Table 2:** Literature Included in the Review

Author(s)	Type	Approach	Method	Theoretical Approach	Personality Perspective
Anglin et al. (2018b)	Reward	Quantitative	Multilevel GLM, multilevel logistic	Social Role Theory	Campaign owner-centered
Bollaert et al. (2019)	Reward	Quantitative	OLS regression	No theory mentioned	Campaign owner-centered
Butticé and Rovelli (2020)	Reward	Quantitative	Probit models	Social Role Theory	Campaign owner-centered
Calic and Shevchenko (2020)	Reward	Quantitative	Logistic and OLS regression	Signaling Theory, Entrepreneurial Orientation	Campaign owner-centered
Davidson and Poor (2015)	Reward	Quantitative	OLS regression	No theory mentioned	Campaign owner-centered
Gera and Kaur (2018)	Reward	Quantitative	Logistic regression	No theory mentioned	Campaign owner-centered
Harburg et al. (2015)	Reward	Qualitative	Semi-structured interviews	Social Cognitive Theory	Campaign owner-centered
Macht and Chapman (2019)	Reward	Qualitative	Not clearly specified	Psychological Capital*	Campaign owner-centered
Rodriguez-Ricardo et al. (2019)	Reward	Quantitative	Structural equation modeling (SEM)	Self-Determination Theory	Investor-centered
Rottler et al. (2020)	Reward	Quantitative	GLM	Socioanalytic Theory	Campaign owner-centered
Ryu and Kim (2016)	Reward	Quantitative	Cluster analysis, ANOVA	Self-Determination Theory	Investor-centered
Shin and Lee (2020)	Reward	Quantitative	Hierarchical regression	No theory mentioned	Investor-centered
Shneor and Munim (2019)	Reward	Quantitative	SEM	Theory of Planned Behavior	Investor-centered
Short and Anglin (2019)	Reward	Quantitative	Multilevel regression, multilevel logistic regression	No theory mentioned	Campaign owner-centered
Sundermeier and Kummer (2019)	Reward	Quantitative	ANCOVA	Dual-Process Theory	Campaign owner-centered
Thies et al. (2016)	Reward	Quantitative	OLS regression	Signaling Theory	Campaign owner-centered
Tseng (2020)	Reward	Quantitative	Partial least squares approach to SEM (PLS-SEM)	Expectation-Confirmation Theory	Investor-centered
Creek (2018)	Reward, Equity	Quantitative	Regression	Social Exchange Theory, Life History Theory	Campaign owner-centered
Leonelli et al. (2020)	Equity	Quantitative	OLS regression	No theory mentioned	Campaign owner-centered
Moritz et al. (2015)	Equity	Qualitative	Semi-structured interviews	Information Asymmetry*	Hybrid approach
Stevenson et al. (2019)	Equity	Quantitative	Path analysis, Chow tests	Control Theory	Investor-centered
Troise and Tani (2020)	Equity	Quantitative	PLS-SEM	Entrepreneurial Decision-Making Theory	Campaign owner-centered
Moss et al. (2015)	Lending	Quantitative	Cox proportional hazards	Signaling Theory	Campaign owner-centered
Netzer et al. (2019)	Lending	Quantitative	Binary logit model	No theory mentioned	Campaign owner-centered
Bernardino and Santos (2016)	Donation	Quantitative	Logistic regression	No theory mentioned	Campaign owner-centered
Rodriguez-Ricardo et al. (2018)	Donation	Quantitative	SEM	Social Identity Theory	Investor-centered
Kim and Hall (2021)	No differentiation	Quantitative	PLS-SEM	Value-Attitude-Behavior Theory	Investor-centered
Kim et al. (2021)	No differentiation	Quantitative	PLS-SEM	Personality Theory	Investor-centered

*Theoretical scaffolding

For the content analysis, we followed the direction of our research question and best practices (e.g., Colombo 2020; Jones et al. 2011; Mochkabadi and Volkmann 2020). We analyzed (1) the contents of the qualitative articles, (2) the results of the quantitative papers focusing on crowdfunding outcomes, and (3) the limitations and future research opportunities suggested by the authors of the reviewed papers.

(1) We summarized the results of the three articles in our sample that utilize a quantitative approach and provide an overview of these within Table 3.

**Table 3:** Summary of Quantitative Results by Crowdfunding Type

Author(s)	Principle Topic	Sample Size and Type	Sampling Procedure	Method Used	Theory Employed
Moritz et al. (2015)	Investor communication	23 semi-structured interviews: 12 investors, 6 new ventures and 5 third parties (mostly platform operators)	Mix of selective and snowball sampling	Exploratory qualitative inductive, theory-building from cases	Information Asymmetry*
Harburg et al. (2015)	Self-efficacy	53 semi-structured interviews	Snowball sampling	Structured quantitative (thematic) analysis (largely deductive)	Social Cognitive Theory
Macht and Chapman (2019)	Self-efficacy	10 crowdfunding campaigns (475 comments)	Semi-random' with cutoff at > = 30 comments	Qualitative interpretive (not clearly inductive)	Psychological Capital*

*Theoretical scaffolding

(2) We examined the subset of twelve quantitative papers focusing on crowdfunding success from our literature selection in more detail. First, for each quantitative study reviewed, we extracted the personality variables examined by the authors. We then supplement these variables with the personality constructs identified within the conceptual background and use them as the basis for our subsequent analysis in Table 4. We examined the findings of the quantitative analysis conducted in detail and extracted all significant and non-significant findings regarding personality variables. Next to these variables the findings were assigned to the crowdfunding type and success variable (e.g., funding success, amount raised, total backers) researched by the authors of the representing article (Table 4). As some authors examine multiple personality variables or different crowdfunding types simultaneously, one article can account for more than one effect displayed in Table 4. As before, one researcher conducted the assignment of the quantitative findings, followed by a review by another researcher and subsequent discussions to eliminate differing assessments.

**Table 4:** Summary of Quantitative Results by Crowdfunding Type

Personality Trait	Success (0/1)				Amount Raised				Number of Backers				Author(s)
	RB	EB	DB	LB	RB	EB	DB	LB	RB	EB	DB	LB	
Big Five													
Openness	↑				↑				↑				Gera and Kaur (2018); Rottler et al. (2020); Thies et al. (2016)
Conscientiousness	↾⇂			↑	↑↾				↑↾				Gera and Kaur (2018); Moss et al. (2015); Rottler et al. (2020); Short and Anglin (2019)
Extraversion	↑↾				↑				↑				Gera and Kaur (2018); Rottler et al. (2020); Thies et al. (2016)
Agreeableness	↑⇂				↑				↑				Gera and Kaur (2018); Rottler et al. (2020); Thies et al. (2016)
Neuroticism	↓⇂				↓				↓				Gera and Kaur (2018); Rottler et al. (2020); Thies et al. (2016)
*Dark Triad*													
Narcissism	↓∩	↓∩			↓∩				↓∩				Anglin et al. (2018b); Bollaert et al. (2019); Butticé and Rovelli (2020); Creek (2018); Leonelli et al. (2020)
Machiavellianism		↓∩			⇂								Creek (2018)
Psychopathy	↓				⇂	↑							Creek (2018); Leonelli et al. (2020)
*Other Traits*													
Self-efficacy					(↑)*								Shneor and Munim (2019)
Innovativeness	↑∩⇂				↑↓∩↾				↑↓∩				Calic and Shevchenko (2020); Moss et al. (2015); Short and Anglin (2019)
Risk-Taking	∩⇂			↑	↑∩⇂				∩⇂				Calic and Shevchenko (2020); Moss et al. (2015)
Autonomy	∩↾			↑	↑∩↾				↑∩↾				Calic and Shevchenko (2020); Moss et al. (2015)

RB (reward-based), EB (equity-based), DB (donation-based) and LB (lending-based)

↑/↓ for linear results; ∩ for inverted U-shaped relations; ↑/ ↓/∩ directions of insignificant results

*Indirect effect via mediator

(3) Next, we closely examined all studies' limitations and the suggested future research identified by the authors of all 28 articles. Hereby, we employed three steps, following a similar approach to that of Jones et al. (2011) for identifying and subsequently coding topic themes. First, we extracted the mentioned limitations and future research sections for each paper. Second, we summarized these sections to reflect their key points (Table 5). One author conducted this step, followed by the mentioned review and discussion process with another researcher. Third, as future research opportunities are of particular interest to the scientific community, we then continued to cluster the mentioned research opportunities into categories. Therefore, two authors independently categorized the future research opportunities mentioned by the respective authors of the reviewed papers, clustering them by similarity (e.g., “We thus advise scholars to extend our work to alternative types of crowdfunding campaigns and platforms.” (Butticé and Rovelli 2020, p. 5) and “future research can be extended to other forms of crowdfunding, such as peer-to-peer lending” (Leonelli et al. 2020, p. 55)). Next we compared the clusters and resolved the remaining differences by reaching consensus between the authors (e.g., splitting the topic “perspectives” into the topics “perspective” and “context”). We next discussed and subsequently assigned topic and subtopic names to the five resulting clusters and twelve subclusters. In many cases, articles reviewed pointed out multiple future research opportunities (e.g., the use of alternate methods and variables, larger samples, etc.). Therefore, we counted some articles into more than one topic cluster (e.g., Butticé and Rovelli (2020) state: “We thus advise scholars to extend our work to alternative types of crowdfunding campaigns and platforms” categorized in our topic “Context” and subtopic “Crowdfunding Type”, but the authors also advise: “replicate our study on a subsample of entrepreneurs administering them the Narcissistic Personality Inventory” categorized in our topic “Methods” and subtopic “Approach” (Butticé and Rovelli 2020, p. 5)). Figure 5 provides an overview of how many of the reviewed articles mentioned one or more of the five future research topics.

**Table 5:** Limitations and Future Research Derived from the Literature

Author(s)	Limitations	Future Research
Anglin et al. (2018b)	Limitations of text based approach Moderating variables effect on narcissism Impression management as thread for the results	Mechanisms linking narcissistic personality and rhetoric Influence of other personality traits Use of narcissism between different demographics Components of narcissism
Bernardino and Santos (2016)	Small sample size Cross-sectional design limits results	More/other dependent variables Influence of personality traits on decision, risk, trust, etc Other countries
Bollaert et al. (2019)	No limitations mentioned	Other crowdfunding models
Butticé and Rovelli (2020)	Only reward-based crowdfunding Reliance of narcissism measurement	Other crowdfunding models Combine with other approaches (questionnaire)
Calic and Shevchenko (2020)	Limitations of text-based approach	Use built dictionary Longitudinal studies Innovativeness in crowdfunding Other crowdfunding models
Creek (2018)	Limited to US data Only successful campaigns Lack of control variables	Test both successful and unsuccessful campaigns
Davidson and Poor (2015)	Small sample size Not a representative sample	Measure by content analyzing Analyze interaction with backers Analyze cultural worker’s attitude Longitudinal data Attitude vs. actual use of crowdfunding
Gera and Kaur (2018)	Limitations of text-based approach	Influence of personality traits on trust, credibility, commitment, intention, etc
Harburg et al. (2015)	No limitations mentioned	Use additional quantitative methods Include more variables Run further controlled experiments
Kim and Hall (2021)	Limited to Korean data Data generated during COVID-19 pandemic Focus on consumers who'd already participated in crowdfunding	Examine the influence of crisis on investments Focus on non-participants of crowdfunding
Kim et al. (2021)	Limited to Korean data Data generated during COVID-19 pandemi	Examine the influence of crisis on investments Employ different research methods (e.g., big data and AI analysis) Future research on the personality of the entrepreneur
Leonelli et al. (2020)	Limited to UK data Only equity-based crowdfunding	Relationship with other forms of finance Other crowdfunding models Other countries Replication
Macht and Chapman (2019)	Extreme cases are not considered qualitative research might be subjective No representative sample Based on secondary data	Investigate in extreme cases Larger sample size Include researches from different backgrounds
Moritz et al. (2015)	No limitations mentioned	Pseudo-personal communication and social medias effect on reducing information asymmetry Extend to other type of crowdfunding Influence of platform business model on communication of the venture Heterogeneity of investors and implications for communication
Moss et al. (2015)	Only perceived not real behavior Limitations of text-based approach Only lending-based crowdfunding Question of practical relevance	Access role of the lender Impact of investments Interaction of counterparties Other regions Focus on the entrepreneurs / their situation
Netzer et al. (2019)	No limitations mentioned	Different populations Other types of unsecure loans Extend to other types of media Extend to other types of industry/behavior
Rodriguez-Ricardo et al. (2018)	Did not specify the type of crowdfunding Perspective of fund seekers or platforms not included	Moderating effect of business type Include all three actors (crowdfunders, fund-seekers and platforms)
Rodriguez-Ricardo et al. (2019)	Specify crowdfunding context Include previous crowdfunding experience Only intentional behavior measured Self-report data	Empirical measures from crowdfunding platforms Combine intrinsic and extrinsic motivation
Rottler et al. (2020)	Decision bias	Investigate in interaction effects Include additional variables (e.g., other signals) Employ multi-method approaches (e.g., qualitative methods, eye-tracking) Narrow-facet level of personality
Ryu and Kim (2016)	Examined only existing sponsors	Effects of cross-network externalities to generate new sponsors Examine factors of sponsor loyalty Typology of crowdfunding creators Interaction between creators and sponsors Preferred project per type of sponsor Relationship between motivation and behavior Examine other platform characteristics and their effect Investigate moderating factors
Shin and Lee (2020)	Survey conducted with college/graduate students Only reward-based crowdfunding Study referred to a well-known platform	Examine various consumer groups Include more control variables (e.g., prejudice, commerce characteristics) Create a consistent questionnaire environment Include other types of crowdfunding
Shneor and Munim (2019)	Generalizability beyond the national and platform Only reward-based crowdfunding Only one method Only self-reports	Longitudinal data Previous (crowdfunding) experience Other crowdfunding models Alternative theoretical frameworks (e.g., technology acceptance model, social capital theory and social cognitive theory
Short and Anglin (2019)	No limitations mentioned	Replications
Stevenson et al. (2019)	No limitations mentioned	Self-efficacy in other entrepreneurial context
Sundermeier and Kummer (2019)	Selection bias	No future research mentioned
Thies et al. (2016)	Limitations of text-based approach Probably not transferable (context specific) No information on the other aspects of text / video Only broader traits Transparency of IBM	Validate in other settings Fine grained classification of personality Combine with other approaches (questionnaire)
Troise and Tani (2020)	Examine other parameters Larger sample size	Relation of self-efficacy in equity crowdfunding to emotions, cognitive parameters, capabilities and environment Larger sample size Replication of the study Use more engaging methodology
Tseng (2020)	Limited to Taiwanese data Selection bias Missing control variables (e.g., residential area, occupation)	Use better sampling techniques

## Results

### Descriptive results

**Fig. 2:**
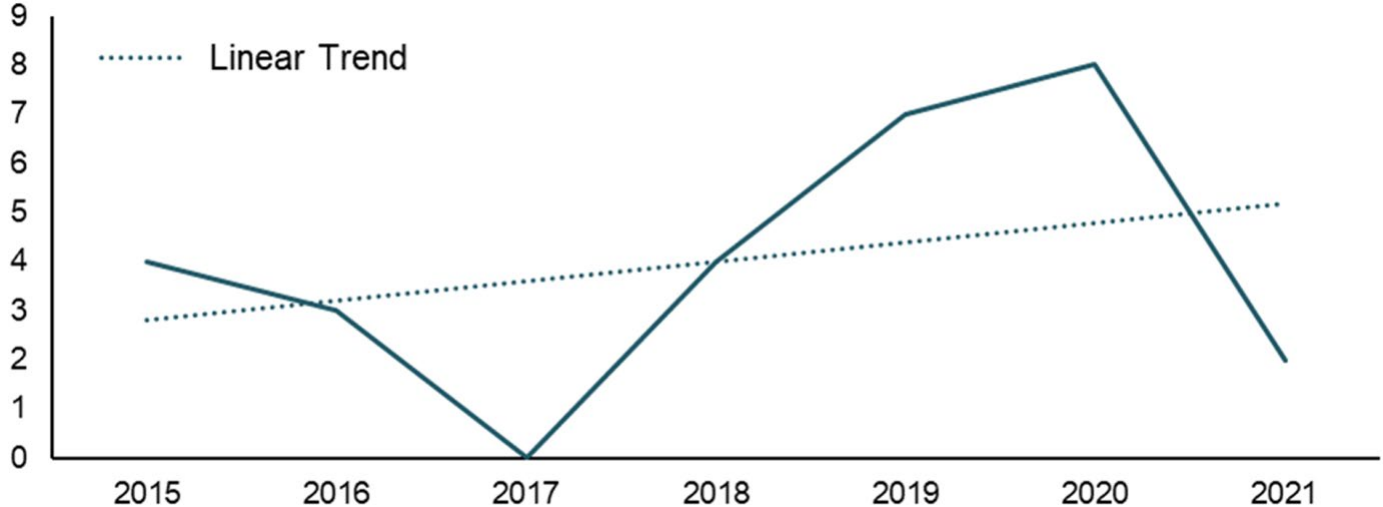
Number of Publications per Year

Our analysis spans 28 articles. These were published between 2015 and March 2021 with a low point of no published papers in 2017 and an increasing trend in more recent years (Fig. 2).

Our search returned papers focusing on the following personality constructs in line with our search terms: the Big Five in general (Bernardino and Santos 2016; Davidson and Poor 2015; Gera and Kaur 2018; Kim and Hall 2021; Kim et al. 2021; Rottler et al. 2020; Ryu and Kim 2016; Thies et al. 2016), only openness (Moritz et al. 2015), only conscientiousness (Moss et al. 2015; Short and Anglin 2019), only extraversion (Netzer et al. 2019) the Dark Triad (Creek 2018; Leonelli et al. 2020), only narcissism (Anglin et al. 2018b; Bollaert et al. 2019; Butticé and Rovelli 2020), self-efficacy (Harburg et al. 2015; Macht and Chapman 2019; Shneor and Munim 2019; Stevenson et al. 2019; Troise and Tani 2020), innovativeness (Calic and Shevchenko 2020; Moss et al. 2015; Rodriguez-Ricardo et al. 2018; Shin and Lee 2020; Short and Anglin 2019; Tseng 2020), and locus of control (Rodriguez-Ricardo et al. 2018). Also, the broad search for the term “personality” in general also revealed additional traits investigated by researchers in the context of crowdfunding: risk-taking (Calic and Shevchenko 2020; Moss et al. 2015; Short and Anglin 2019), autonomy (Moss et al. 2015; Short and Anglin 2019), as well as charisma and hubris (Sundermeier and Kummer 2019). The crowdfunding literature does not yet reflect the term “need for achievement” as a personality construct.

**Fig. 3:**
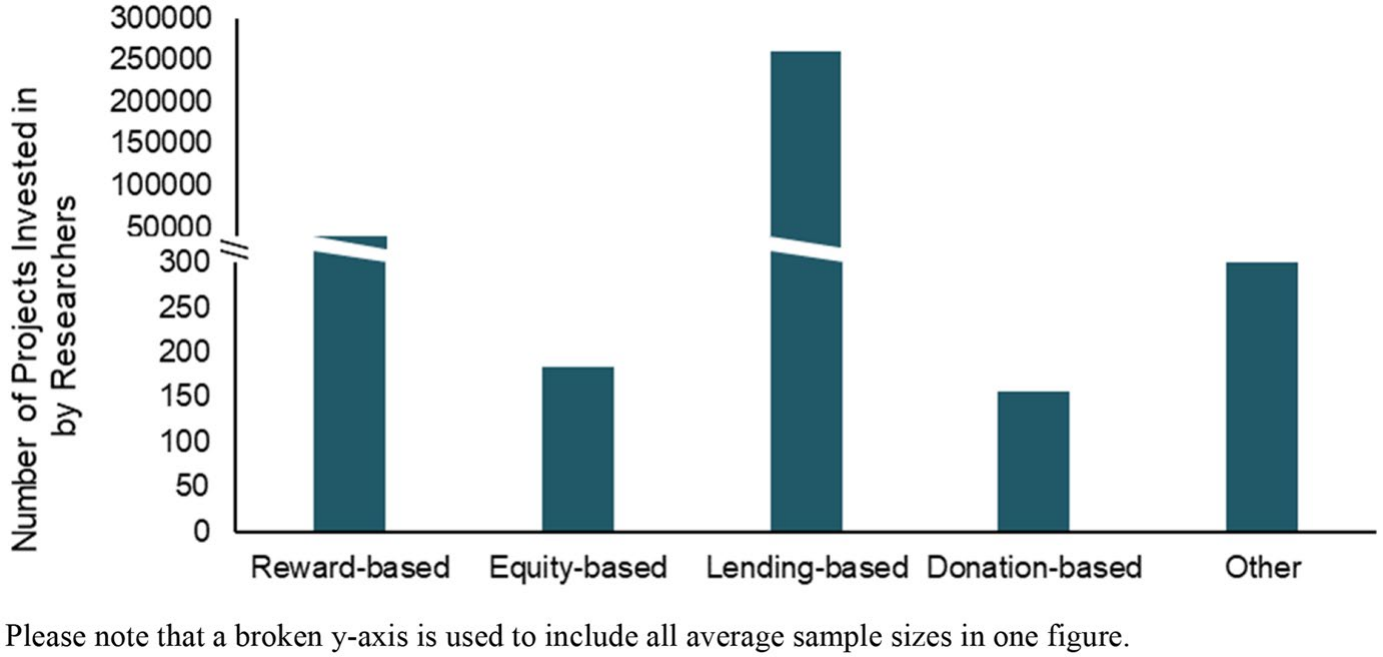
Average Examined Campaigns per Crowdfunding Type

In 17 of these articles, authors primarily investigate personality aspects in reward-based crowdfunding rather than in other crowdfunding types (Table 2). This trend might be due to the easy accessibility of Kickstarter data via openly available tracking platforms such as Kickspy. It is also noteworthy that both reward- and lending-based crowdfunding permit the authors to use larger samples of campaign data (on average) compared to donation-based and particularly equity-based forms of crowdfunding (Fig. 3).

**Fig. 4:**
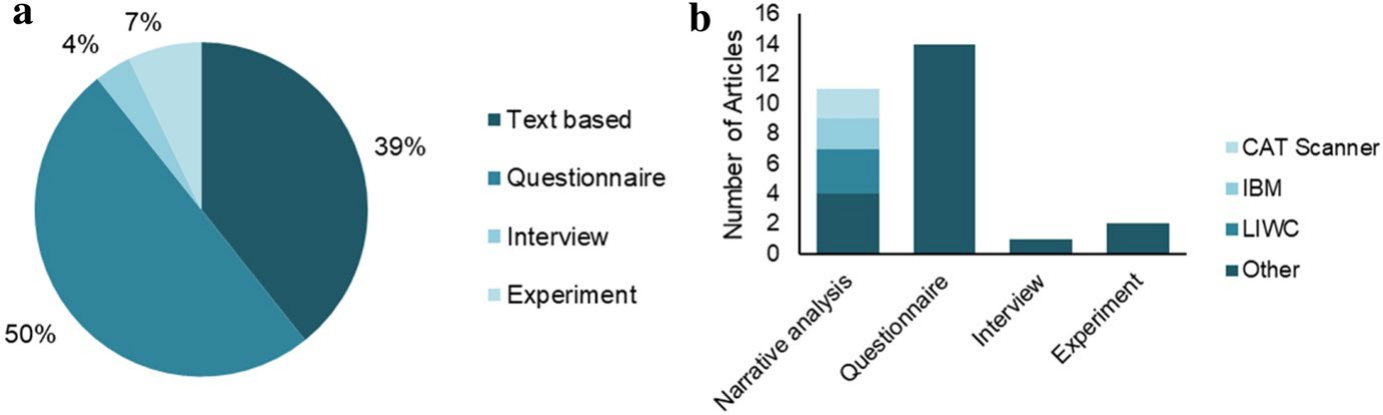
Approach: Percentage Distribution and Investigation Method

The methods used within the selected papers are based on questionnaires, narrative analysis, experiments, and interviews (Fig. 4a). Most of the articles are based on methods that focus on questionnaires or the text of a given campaign. The software tools most frequently employed for narrative analysis conducted in 11 articles are Linguistic Analysis and Word Count (LIWC) and CAT Scanner. Further, two authors used the artificial intelligence based tool IBM Personality Insights (Fig. 4b).

Of the 28 articles, only three base their research on qualitative approaches. These conducted semi-structured interviews in two cases (Harburg et al. 2015; Moritz et al. 2015) and in the third case coded comments on crowdfunding pages regarding e.g., moral support provided by the investors (Macht and Chapman 2019). The remaining articles follow a quantitative approach largely based on regression models (Table 2).

The authors of the articles selected for our review employ a number of theories. Three articles base their research on Signaling Theory (Spence 1978). Social Role Theory (Eagly and Wood 2012) and Self-Determination Theory (Deci and Ryan 2008) were also used by more than one author team. Additional theories utilized in the articles can be derived from Table 2.

Regarding the perspective taken in the articles, across all 28 studies, 18 focus on the entrepreneur’s or campaign creator’s view. Nine articles take the investor perspective. Strikingly, only one author team took a more comprehensive approach (Moritz et al. 2015) by investigating all parties involved: the entrepreneurs, investors, and any third parties involved, e.g., platform representatives (Table 2).

### Results of the thematic analysis

For a more in-depth thematic analysis, we set three priorities. First, we summarized the results of the three qualitative studies. Second, we categorized previous quantitative studies in a way that can be easily utilized by future authors. Third, we summarize and categorize what other authors consider to be the essential future research steps in personality research on crowdfunding.

#### Summary of the qualitative articles reviewed

Three out of the 28 research papers within this literature review are qualitative in nature (Table 3). First, the qualitative-empirical study of Moritz et al. (2015) inductively investigates the role of investor communication as a medium for overcoming information asymmetries. Therefore, the authors conducted 23 interviews with investors, representatives of new ventures, and third party stakeholders such as platform operators. The study finds that within the crowdfunding process, personal communication is replaced by pseudo-personal communication via the Internet and that communicating soft personality factors, e.g., openness is vital to reduce perceived *information asymmetry*, i.e., when one party has more (private) information than the other. In so doing, the authors took the perspective of different participants in the crowdfunding process and thereby provided the only paper that simultaneously investigates multiple perspectives and goes on to build theory from cases.

Second, Harburg et al. (2015) investigate the influence of crowdfunding ecosystems on the entrepreneurs' self-efficacy. The authors thereby conducted 53 semi-structured interviews and rely on Bandura’s *social cognitive theory* (Bandura et al. 1999)– which maintains that people’s knowledge acquisition is based on observing others in social context and the media. Therefore, the study is clearly deductive in nature. The authors report that entrepreneurs gain self-efficacy via the received feedback and number of backers supporting them, metrics showing their progress on the funding page, and examples of succeeding entrepreneurs. Nevertheless, the entrepreneurs' self-efficacy can also decrease when facing a lack of public validation or their project fails in front of the crowd (e.g., experiencing shame).

Third, Macht and Chapman (2019) also examine self-efficacy supplemented by other psychological capital aspects like optimism and resilience in the context of crowdfunding. Their qualitative interpretative work investigates the associations between the crowds' comments within a given campaign and fund seekers' human, social, and psychological capital. By coding and thematically analyzing 475 comments from ten crowdfunding campaigns (examining only those with a minimum of 30 comments in a selection process that can at best be described as semi-random), the authors core finding is that the crowd can increase the entrepreneurs' self-efficacy, hope, optimism, and resilience by providing support and by showing support and criticism within their comments. The generalizability of this finding is limited, given the moderate sample size. Also, the methodology used is not clearly specified and it is unclear if this work is inductive or rather a more deductive approach that begins with psychological capital and goes on to “test” this qualitatively.

With the exception of the study of Moritz et al. (2015), the qualitative studies focus not on the personality displayed within the crowdfunding process but on gaining self-efficacy via the crowdfunding process itself. While the degree to which an individual’s personality can change through a single crowdfunding campaign may be questionable, these studies focus on an angle of personality in crowdfunding that has clearly been neglected by the other studies within this literature review. Thereby, such qualitative studies can help explore future research avenues not yet represented in the body of literature.

#### Categorization of results of the quantitative articles reviewed

Only twelve articles quantitatively analyze the effects of personality on campaign outcomes. We focus on the independent personality variables reflected by the papers retrieved in our literature search. The outcome of a campaign is measured either by a dummy variable for success (goal reached yes/no), the actual amount raised (a continuous variable), the number of contributors to a campaign (as a count variable), or a combination of these three.

Three articles study the Big Five traits (Gera and Kaur 2018; Rottler et al. 2020; Thies et al. 2016) and two additional studies examine the single Big Five trait conscientiousness (Moss et al. 2015; Short and Anglin 2019). The authors find strong evidence for a positive impact of openness on crowdfunding success and suggest a positive influence of agreeableness and extraversion and a negative influence of neuroticism (Gera and Kaur 2018; Rottler et al. 2020; Thies et al. 2016). It is noteworthy that for most Big Five factors, the authors do not report similar findings, but find both significant and non-significant effects. Only openness and its positive influence on campaign success in reward-based crowdfunding seems to be a robust relationship across the quantitative studies reviewed (Table 4).

Focusing on the Dark Triad, we see that while existing results for other crowdfunding types often contradict each other, in some cases there are clear tendencies, such as for the negative but inverse u-shaped effect of narcissism on crowdfunding success (even across different measures of success). Although the articles report no significant results for Machiavellianism, they report some evidence for the effects of psychopathy. For example, Creek (2018) finds a positive relationship between the amount raised and psychopathy in equity-based crowdfunding, contrary to the opposite finding of Leonelli et al. (2020) regarding campaign success.

Finally, we report our findings on the study of the additional (frequently used) personality traits within the identified crowdfunding literature. First, Shneor and Munim (2019) find an indirect effect of self-efficacy in reward-based crowdfunding, in particular a significant influence on their mediator variable “financial contribution intention”. Second, Short and Anglin (2019) find a significant negative effect of innovativeness on the amount raised, and Calic and Shevchenko (2020) find positive but also significant inverted u-shaped relations for innovativeness in all three crowdfunding performance measurements (success, amount raised, and number of backers). Both studies were conducted in a reward-based crowdfunding setting. Third, some authors find that risk-taking entrepreneurs succeed more often in lending-based crowdfunding campaigns (Moss et al. 2015), while Calic and Shevchenko (2020) report inverted u-shaped relationships between risk-taking and campaign success in reward-based crowdfunding. Further, it is noteworthy that, while risk-takers are more likely to receive crowdfunded loans, they are less likely to succeed with other types of crowdfunding. Fourth, autonomy negatively affects the amount raised in reward-based crowdfunding (Short and Anglin 2019) and shows an inverted u-shaped relation across all performance measurements (Calic and Shevchenko 2020). In lending-based crowdfunding, however, Moss et al. (2015) report a positive effect of autonomy.

#### Analysis of the Future Research Sections

**Fig. 5:**
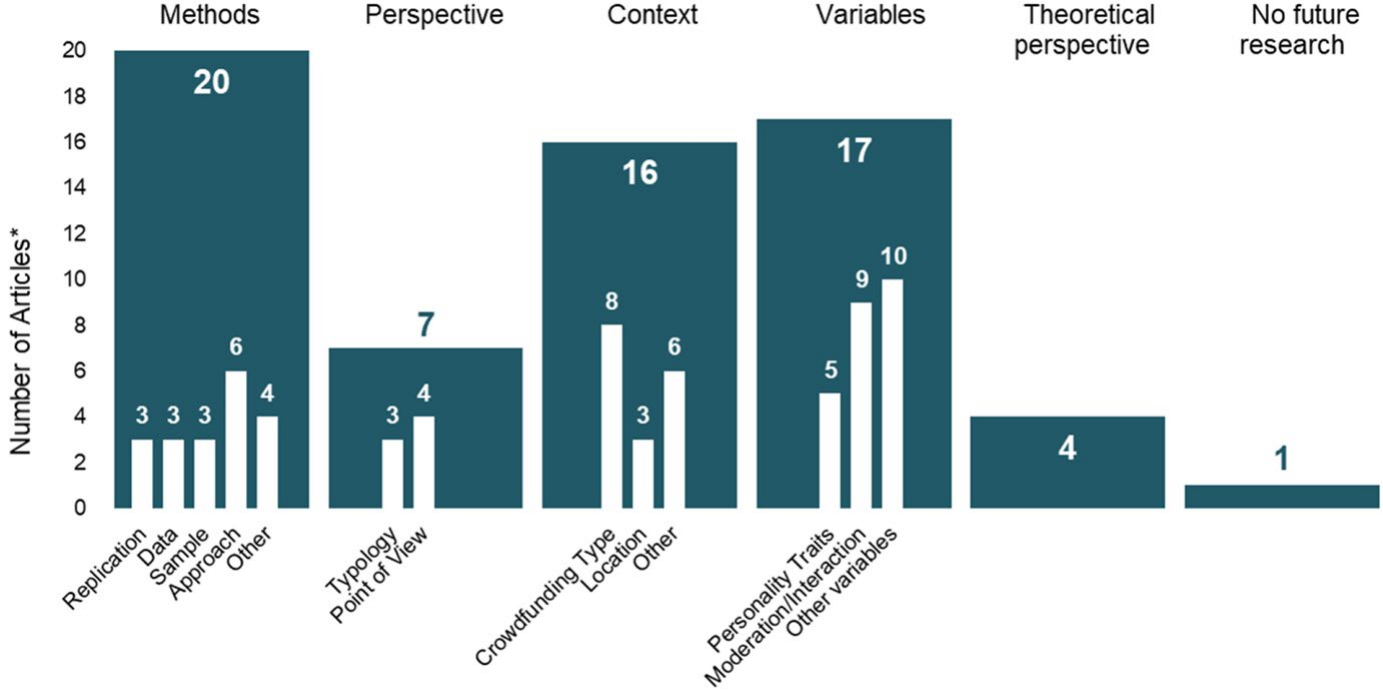
Future Research Suggestions from the Articles Reviewed categorized in Topics and Subtopics. *Number of articles in a subtopic may add up to more than the number of articles within a topic as some articles point out multiple future research opportunities (e.g., the use of alternate methods and variables, larger samples, etc.)

The analysis of the critical gaps for future research in personality and crowdfunding is based on all 28 articles included in the literature review. Table 5 provides detailed insights into what the representative authors identified as limitations in their articles and how they would like to see future research evolve to address these concerns. We summarize, categorize and quantify the individual statements in Fig. 5.

Overall, we found that first, the authors call for future studies that employ more comprehensive methods (e.g., other approaches or larger sample sizes). Second, the inclusion of more variables is important for the authors to reduce omitted variable bias and endogeneity concerns. Many of them suggest including not only additional controls, but further constructs such as trust, credibility, commitment, and intention (Gera and Kaur 2018). Third, nearly equally frequently, authors request future authors to the transfer their analysis to other contexts, such as to other types of crowdfunding. Sixteen articles mentioned this aspect, whereby eight specifically refer to shifting the focus from one crowdfunding type to another. Finally, other ideas for future research identified across the articles are: a change of perspective, for example by investigating other stakeholders, and the inclusion of other theories, e.g., Social Capital Theory or Social Cognitive Theory (Bandura et al. 1999; Shneor and Munim 2019).

## Discussion

Personality is an important and under-researched topic in entrepreneurial finance, especially in the crowdfunding context, expressed in a growing body of research that has peaked in 2020. In this literature review, we retrieved articles focusing on nearly every personality construct included in the search terms (except for the “need for achievement”). Further, the more generalized search term “personality” uncovered additional personality constructs, which are risk-taking (Calic and Shevchenko 2020; Moss et al. 2015), autonomy (Calic and Shevchenko 2020; Moss et al. 2015), and traits associated with charisma and hubris (Sundermeier and Kummer 2019). Risk-taking describes the tendency to make risky decisions in the presence of uncertainty (Knight 1921); autonomy stands for the need for independence. Charisma and hubris combine personality traits attributed to entrepreneurs, such as excessive pride and self-confidence (hubris) or charm and persuasion (charisma) (Sundermeier and Kummer 2019).

We further find that within studies that focus on the Dark Triad, more studies cover narcissism than psychopathy or Machiavellianism. This difference could be rooted in the relatively high salience of the narcissism construct in narratives relative to the other traits. However, the popular and well-known measurement of narcissistic rhetoric introduced by Chatterjee and Hambrick (2007), while measuring CEO narcissism, might also be why many researchers focus on this trait.

### Gaps and future research

In the following, we discuss key findings from our results in order of importance. We thereby not only examine the results of the quantitative articles included in the analysis of personality effects on crowdfunding performance, but combine these with the literature gaps identified by all articles included in the review. Therefore, we take a closer look at personality traits as non-linear, the use of narrative analysis methods, the context dependency of personality research in crowdfunding, and the specific personality perspective taken by the authors.

#### Personality as non-linear

Apart from the rather consistent results for openness and narcissism, the results differ from article to article and show no consistent pattern (Table 4). However, it is important to mention the inverted u-shape that authors often find for several personality traits. Miller (2015) argues convincingly that personality attributes are Janus-faced and that the negative aspects of the entrepreneurial personality have been largely ignored so far. Similarly, Calic and Shevchenko (2020) conclude that personality components such as innovativeness or risk propensity can be perceived as desirable by investors to a certain degree, but lose their positive appeal when over-expressed and hence are subject to a threshold effect. Although such nonlinear relationships appear to make sense when investigating personality in a complex context like crowdfunding, only a few authors analyze nonlinear relationships (e.g., quadratic relations) and surprisingly none mention this approach as potential for future research. We nevertheless argue that future research must pay special attention to these findings by testing for or including quadratic terms when examining personality effects in crowdfunding. A research question focusing on this non-linear relationship could entail: *Do personality traits displayed in crowdfunding campaigns reach a saturation point at which they are overexpressed and consequently diminish the engagement/contribution level of the crowd?* Answering this question would resolve inconsistencies in the current literature and fill a research gap regarding potentially underexplored quadratic effects of expressed personality in crowdfunding. Further, it would contribute to research on the effects of perceived personality expressions on impression formation (Hamilton et al. 1980). In practice, answering this question would also help crowdfunding entrepreneurs evaluate campaign material (e.g., videos) in a more nuanced way.

#### Use of different methods

Eleven of the studies examined base their research on software-based text analysis methods which are increasingly popular in entrepreneurship research, particularly so in studies related to personality. The perks are undeniable: employing this method facilitates access to larger samples that were not previously accessible. Using these methods, researchers rely on publicly available online text snippets such as letters to shareholders, IPO prospectuses, tweets, campaign page text, and even transcribed voice and video recordings, e.g., manager earning calls (Aerts and Yan 2017; Golbeck et al. 2011; Harrison et al. 2019; Loughran and McDonald 2013). However, the disadvantages of such methods should not be underestimated. On the one hand, there is the problem of validity. The methods employed are often validated only based on self-written imaginary text, generated in experimental settings and not on topic-specific text with an economic focus (Mairnesse et al. 2007; Pennebaker and King 1999). On the other hand, campaign pages’ texts are not necessarily authored by the entrepreneurs themselves, although assumed by this method of text-based personality assessment. It is also possible, that third parties such as public relations firms are hired to craft the campaign text on behalf of the entrepreneur or startup team. Analyzing these campaign texts, we must question whether the traits measured actually capture the campaign creator's personality.

So what do these studies actually measure? Some authors argue that they might have measured *perceived* personality rather than the entrepreneurs' true personality (Moss et al. 2015). Often, researchers are simply interested in the impact of personality traits as perceived by investors on crowdfunding success and do not require knowledge about the true underlying personality of the entrepreneur. As long as the studies find a correlation between the measured construct and crowdfunding success, the results suggest that the method is functioning as intended. Also, perceived personality could be a valid measure for a number of research questions, because investors are limited to the information presented on the campaign page. For instance, this could be the case for big data researchers or in entrepreneurial finance (Harrison et al. 2019), but may not be the case for psychologists that study personality in more personal context (Bozionelos 2017). In cases where the true personality of an entrepreneur is needed to answer a particular research question, text-based methods along with the stated limitations regarding perceived personality could present a real challenge. Future research could tackle this issue by combining, different methods such as combining text-based methods with psychological questionnaires as argued by Butticé and Rovelli (2020). Also, other studies analyzed within this paper highlight the need for the use of different methods while investigating personality in the crowdfunding context (see Table 5). Letting some of these authors speak for themselves they “encourage future researchers in crowdfunding to analyze empirical measures from crowdfunding platforms” (Rodriguez-Ricardo et al. 2019, p. 12), argue that “qualitative and quantitative tools” (Davidson and Poor 2015, p. 303) are needed in this research area, and emphasize that including e.g., questionnaires in their research model “would contribute to add reliability to our study and to rule out possible alternative explanations” (Butticé and Rovelli 2020, p. 5). An unanswered research question focusing on the combination of different personality measurements, therefore, is: *Does a narrative analysis of crowdfunding campaign texts reveal similar personality trait expressions as validated personality questionnaires conducted by the campaign owners?* Research focusing on this question could contribute to the ongoing debate on the effect of individual-level attributes of the entrepreneur on campaign success. Revealing if the effect of perceived personality outweighs the effect of inner personality (or vice versa) in terms of venture financing success in crowdfunding could monumentally influence crowdfunding practice as entrepreneurs can shape their narratives, and by extension, their impressions on people, but their internal personality is more or less fixed (Costa and McCrae 1988).

#### Context dependence

Due to the newness of the crowdfunding research field and the use of highly recent methodologies still under development, there are few studies in general and even fewer replication studies in this area. Only one article intentionally replicates the work of another author team (Short and Anglin 2019). In their article, the authors conclude that “individuals should exercise extreme caution in regard to assuming that findings in one context can be generalized to others” as they “failed to replicate any of the hypotheses where the authors originally found support” in one of the included replication studies (Short and Anglin 2019, p. 12). This comment by Short and Anglin (2019) is strikingly similar to what we actually observe in our review of studies in this field. Trying to summarize the relationships tested by the quantitative studies on personality and crowdfunding campaign success does not result in a clear picture (see Table 4). Instead, many studies find no effects, where others find effects or even contradictory results (e.g., Creek 2018; Leonelli et al. 2020).

One reason for this could lie in the different settings of the studies. Short and Anglin (2019) replicated the study by Moss et al. (2015) in a reward-based crowdfunding context whereas it was initially conducted with lending-based crowdfunding data. With this change in settings there is are also implicit changes in the basic features of the investigated construct, such as investor motivation. For example, while investors in reward-based crowdfunding are often assumed to be intrinsically motivated, investors in other crowdfunding types might behave differently (Cholakova and Clarysse 2015).

Further, it is somewhat puzzling why studies that measure the same constructs in similar settings obtain different results. For example, even in studies conducted in the same setting, e.g., reward-based crowdfunding and studying the same relationship, e.g., between perceived Big Five personality traits of entrepreneurs and campaign success and on the same platform (often Kickstarter), the results can differ (Gera and Kaur 2018; Thies et al. 2016). Although addressing a similar research question, there are striking differences in the methodologies of the full paper by Thies et al. (2016) and the short paper by Gera and Kaur (2018). First, the text used for the calculations in Thies et al. (2016) included the campaign text and the campaign description separately with similar results. On the other hand, Gera and Kaur (2018) use campaign descriptions and profile descriptions from the campaign owners. Second, whereas Thies et al. (2016) base their analysis on a regression model, Gera and Kaur (2018) (although mentioning logistic regressions) report only correlations as results. Third, Thies et al. (2016) analyze 33,420 campaign texts and 12,859 video transcripts, while Gera and Kaur (2018) do not include videos but instead opted to analyze a smaller number of 4059 campaign descriptions and 1721 creator profiles. Fourth, both author teams include different control variables in their analysis. Fifth, using a different time period to obtain the data and regulatory changes could cause systematically different results (Pollack et al. 2021). The example of these two papers (Gera and Kaur 2018; Thies et al. 2016), which appear similar at first, illustrates the problems that future researchers could solve by conducting replication studies. It is undeniable that personality constructs affect crowdfunding outcomes, but since the strength of the influence depends on the circumstances, researchers must pay particular attention to such details.

Therefore, we think that replication studies are particularly important for future research to determine differences in the effects of personality. First, replications are needed across types of crowdfunding and different platforms to observe the effect of this contextualization. This point was made by eight articles included in this research (Fig. 5; e.g., Bollaert et al. 2019; Leonelli et al. 2020) Second, even when the type of crowdfunding and platform are held constant, such replication studies are crucial to generate a reliable knowledge base about the relationships between personality constructs and crowdfunding outcomes. Third, as cultural and geographic factors could also influence crowdfunding outcomes, authors should consider including different regions in their studies as suggested by Bernardino and Santos (2016) and others (Table 5). A specific research question is: *Which context-dependent variables moderate the effects of personality on crowdfunding?* Answering this question could change how entrepreneurial science sees crowdfunding in that the role of personality could illustrate how the different types of crowdfunding might differ from each other more than they do from other forms of venture finance. Entrepreneurial displays of agreeableness to an audience of equity crowdfunding investors could have more implications for angel investments or IPOs than for reward-based crowdfunding and thereby open the opportunity for researchers to transfer findings from the accessible crowdfunding context to more traditional investment settings. Also, the scientific community could learn more about the role of individual crowdfunding platforms within a given type of crowdfunding (e.g., StartEngine and Wefunder for equity crowdfunding) in shaping the effect of individual characteristics like personality on campaign outcomes. Finally, we could also learn more about the role of national culture or geographic context in shaping how personality factors leading to crowdfunding success. This knowledge could help entrepreneurs who are thinking about entering new markets or expanding across borders.

#### Change of personality perspective

In the literature reviewed, we see a focus on studying the personality of the entrepreneur who is assumed to be the campaign creator. Studies on investors' personality, on the other hand, are less frequently conducted, even though there are relatively easy to investigate by survey studies while entrepreneurs are more difficult to access directly regarding their personality (Hambrick and Mason 1984). Studies on investors' personality typically use inventory-based questionnaires (Rodriguez-Ricardo et al. 2019; Shneor and Munim 2019), but have so far neglected studying investor comments for example. There have, however, been studies that investigate investor comment sentiment (Wang et al. 2018) which seems to be leading in a fruitful direction.

Only a few of the articles reviewed focus on the investor personality perspective. They find that social identification with the crowdfunding community and the individual level of innovativeness, unlike internal locus of control, positively affect the intention to participate in crowdfunding (Rodriguez-Ricardo et al. 2018, 2019). Further, Ryu and Kim (2016) categorize crowdfunders into four groups (angelic backers, reward hunters, avid fans, tasteful hermits) employing various factors including the Big Five personality traits, whereas Shneor and Munim (2019) find differences in self-efficacy between investors that contribute higher vs. lower amounts to campaigns.

Only one article by Moritz et al. (2015) includes more than one personality perspectives (e.g., investor, entrepreneur, involved third parties such as platforms). In their qualitative study, they investigate how information asymmetries within the crowdfunding process can be reduced by communication (e.g., of soft factors) between the parties involved via the internet (Moritz et al. 2015). Nevertheless, the authors of the analyzed articles also recognize the potential that arises from investigating other perspectives (Table 5). They argue that future research “should consider the role that [all actors (crowdfunders, fund seeker and platforms)] play in this new phenomenon” (Rodriguez-Ricardo et al. 2018, p. 178) and that it is important to “further analyze the relationship between lender characteristics and those of borrowers” (Moss et al. 2015, p. 47).

Including several perspectives is a promising task for future research. As the saying “Birds of a feather flock together” implies, people that share specific characteristics get along better. In his paper on homogeneity, Marsden (1988) discovers that people that have strong social relations are more likely to share similar attributes. Transferring this idea to the crowdfunding context, Venturelli et al. (2020) investigated the effects of ethnic and gender similarities between investors and entrepreneurs and the positive impact on funding in equity-based crowdfunding. Oo et al. (2019) focus on the mediating effect of similarity (in-group favoritism) between entrepreneurs and investors in reward-based crowdfunding. Additionally, Burtch et al. (2014) found that crowdfunders prefer culturally similar and geographically proximate fund-seekers. Lin and Viswanathan (2016) refer to this phenomenon as “home bias”. Similarly, Mollick (2014) suggests that geography may play an important role. These studies demonstrate the importance of investigating the relationship between funding seekers and investors in the crowd. Therefore, we strongly encourage research on the personality of all parties involved in the crowdfunding process and especially the interaction between investors’ and entrepreneurs’ personality. A concrete research question dealing with this change of perspective is: *Are there interactions between the personalities displayed by entrepreneurs and those of the contributing investors in the crowd?* Answering this question could impact how entrepreneurs approach investors in the crowd. It would also shed light on investors' selection processes when finding crowdfunding campaigns to invest in.

### Implications

Our results have a number of implications for research and practice. First, our study implies that quantitative crowdfunding researchers should pay particular attention to the type of crowdfunding, the measure of success utilized and the selected personality traits when designing their studies. Second, the mixed results for many traits imply a strong need for replication studies to validate the results and methods used. Third, authors should consider qualitative and mixed-methods approaches in future studies to advance and deepen our theoretical knowledge and not just test existing knowledge or theory. Fourth, personality researchers, our results imply that many of these constructs may not be fully distinctive from one another or optimally measured in crowdfunding by using narrative approaches alone. Therefore, it could be helpful to combine different types of analysis to better capture personality traits (e.g., the analysis of campaign text narratives with the analysis of pitch videos, observer ratings or questionnaires). Finally, our results can feed into big data approaches and into studies on deception in crowdfunding and other forms of entrepreneurial finance (e.g., Siering et al. 2016; von Selasinsky and Isaak 2020).

Our study also has several practical implications. First, for entrepreneurs seeking capital from the crowd, our results imply that displaying certain types of personality when crafting their campaign narratives (e.g., openness) in certain types of crowdfunding (e.g., reward-based) can indeed impact the success of their campaign (see Table 4). Entrepreneurs that display openness are presumably more likely to be perceived as having the necessary networking capabilities to succeed with a startup venture.

Second, by examining the results in comparison, investors in the crowd could screen campaigns for traits in which entrepreneurs display personality that improves (or reduces) the probability of a successful outcome, guiding their investment decision beyond just utilization of hard facts (e.g., the number of backers so far and the amount collected so far). Third, crowdfunding platforms could add personality screening inventories when conducting their project due diligence when evaluating project risks (together with other existing factors such as screening for typos and completeness of the campaign text and multimedia) to better pick the winners and improve their preselection of which projects are allowed to enter the crowdfunding process.

### Limitations

Our study also has a number of limitations. First, due to the specialized nature of the subject which requires interdisciplinary approaches, our review covers only a limited number of articles. Second, which factors should be considered as personality traits in a narrower sense is not always clear. We included those which are mostly unquestioned in psychology (particularly the Big Five and the Dark Triad traits) and a number of additional traits that are frequently used in studies that appear in top entrepreneurship journals (e.g., ETP, JBV, etc.) in our literature review (Costa Jr and McCrae 1995; Paulhus and Williams 2002; Rauch and Frese 2007). Nonetheless, this could be further extended by incorporating studies on what some psychologists now refer to as the sixth basic component of personality (the Honesty-Humility trait, yielding the Big Six, also known under the acronym HEXACO) (Ashton and Lee 2007; Saucier 2009). Third, researchers often refer to other psychological constructs while investigating entrepreneurial behavior. These include passion, which describes a strong inclination towards a specific activity (Murnieks et al. 2014) and altruism, i.e., prosocial behavior (Batson and Powell, 2003). Although passion is more of an emotional (Anglin et al. 2018a; Avey et al. 2008) and altruism is more a motivational construct (Rushton et al. 1981) than a personality trait, further research could investigate both in the context of crowdfunding. While including these would have been out of the scope of this study, in an additional informal screening of such literature, we found very few such studies, highlighting a significant research gap regarding plurality of actor perspectives when examining crowdfunding and personality.

### Conclusion

We conclude our literature review on personality research in crowdfunding by noting that this is a very young and budding research field, which still offers considerable room for further research. Our results question a finding of the article “How Should Crowdfunding Research Evolve” that reports no interest by leading editors surveyed in the research field of ‘personality theories’ in crowdfunding (McKenny et al. 2017). Recently, however, we observe an increase in published studies in this research field which indicates growing interest by the scientific community. Newly available analysis methods might be driving this trend. For example, scraping techniques have evolved to more easily gather online data; also, new software tools such as those based on artificial intelligence capitalize on big data approaches and permit the investigation of personality in novel ways.

By identifying crucial gaps in the literature for future research and by highlighting which approaches are needed for this research stream to evolve our review contributes to research on crowdfunding and personality (e.g., Anglin et al. 2018a; Moss et al. 2015) and to research on the entrepreneurial personality more generally (e.g., Kets de Vries 1977; Rauch and Frese 2014). First, future studies should examine non-linear relations between expressed personality traits and crowdfunding success, as personality traits are not dichotomous and can cause different behavior depending on the intensity of expression. Second, there is a need for studies that employ different methods such as mixed-methods approaches to validate narrative analysis techniques with, for example questionnaires or experiments. Third, to obtain a clear picture of personality effects in crowdfunding, replication studies in similar and different contexts are of crucial importance to this scientific field. Fourth, our review revealed that a plurality of personality perspectives would strengthen future research. We hope that our review article will help to encourage research in this area and provide researchers with a first systematic overview of the field.
